# Quantum-Mechanical Study of Nanocomposites with Low and Ultra-Low Interface Energies

**DOI:** 10.3390/nano8121057

**Published:** 2018-12-15

**Authors:** Martin Friák, David Holec, Mojmír Šob

**Affiliations:** 1 Institute of Physics of Materials, Academy of Sciences of the Czech Republic, Žižkova 22, CZ-616 62 Brno, Czech Republic; mojmir@ipm.cz; 2 Department of Materials Science, Montanuniversität Leoben, Franz-Josef-Strasse 18, A-8700 Leoben, Austria; david.holec@unileoben.ac.at; 3 Department of Chemistry, Faculty of Science, Masaryk University, Kotlářská 2, CZ-611 37 Brno, Czech Republic; 4 Central European Institute of Technology, CEITEC MU, Masaryk University, Kamenice 5, CZ-625 00 Brno, Czech Republic

**Keywords:** MoSi_2_, WSi_2_, TaSi_2_, NbSi_2_, elasticity, ab initio, interface energies, Fe_3_Al, disorder

## Abstract

We applied first-principles electronic structure calculations to study structural, thermodynamic and elastic properties of nanocomposites exhibiting nearly perfect match of constituting phases. In particular, two combinations of transition-metal disilicides and one pair of magnetic phases containing the Fe and Al atoms with different atomic ordering were considered. Regarding the disilicides, nanocomposites MoSi${}_{2}$/WSi${}_{2}$ with constituents crystallizing in the tetragonal C11${}_{b}$ structure and TaSi${}_{2}$/NbSi${}_{2}$ with individual phases crystallizing in the hexagonal C40 structure were simulated. Constituents within each pair of materials exhibit very similar structural and elastic properties and for their nanocomposites we obtained ultra-low (nearly zero) interface energy (within the error bar of our calculations, i.e., about 0.005 J/m${}^{2}$). The interface energy was found to be nearly independent on the width of individual constituents within the nanocomposites and/or crystallographic orientation of the interfaces. As far as the nanocomposites containing Fe and Al were concerned, we simulated coherent superlattices formed by an ordered Fe${}_{3}$Al intermetallic compound and a disordered Fe-Al phase with 18.75 at.% Al, the α-phase. Both phases were structurally and elastically quite similar but the disordered α-phase lacked a long-range periodicity. To determine the interface energy in these nanocomposites, we simulated seven different distributions of atoms in the α-phase interfacing the Fe${}_{3}$Al intermetallic compound. The resulting interface energies ranged from ultra low to low values, i.e., from 0.005 to 0.139 J/m${}^{2}$. The impact of atomic distribution on the elastic properties was found insignificant but local magnetic moments of the iron atoms depend sensitively on the type and distribution of surrounding atoms.

## 1. Introduction

Ever increasing demand for energy-conversion units exhibiting a higher efficiency leads to increasing operating temperatures in these systems and, therefore, new materials, which would sustain such conditions, are needed. Because the development of these materials is highly complex and multi-faceted, combinations of often mutually-conflicting properties are rarely found in a single-phase structures. Composites then represent a critically important class of materials. In particular, coherent nanocomposites require optimum matching of constituting phases for their stability and rather low interface energies. In our study, we addressed three nanocomposites combining materials intended for high or elevated temperature applications: two pairs of transition-metal disilicides that are predicted to possess ultra-low interface energies and then a pair of two different phases from the Fe-Al system, which are expected to have the interface energies ranging from ultra low to low values.

Regarding the transition-metal silicides, they are currently considered as very promising bases for future high-temperature structural materials (see, e.g., Refs. [1,2,3,4]), in particular for operational temperatures above those of Ni-based superalloys. At high temperatures, transition-metal silicides are known to combine the ductility and thermal conductivity of metals with high strength and corrosion resistance of ceramics. As far as composites combining them are concerned, MoSi${}_{2}$/WSi${}_{2}$ composite powders with different phase compositions are fabricated via a self-propagating high-temperature synthesis (SHS) method [5]. This approach is widely recognized as an effective manufacturing strategy for the fabrication of materials applied in high-temperature fields, in particular for refractories such as transition-metal carbides, nitrides, silicides, and borides [6]. Preparation of MoSi${}_{2}$/WSi${}_{2}$ composites using elemental powders of Mo, W and Si by the thermal explosion mode of SHS have been theoretically calculated and investigated by experiments in Ref. [7]. Phase composition and microchemical area analyses were conducted by XRD, SEM and EDAX methods. Pure MoSi${}_{2}$/WSi${}_{2}$ composites are fabricated by the thermal explosion mode of SHS, and MoSi${}_{2}$/WSi${}_{2}$ exists as a solid solution of (Mo${}_{x}$,W${}_{1-x}$)Si${}_{2}$ but the chemical elements inside of individual grains are not uniformly distributed. As an alternative processing route, five kinds of WSi${}_{2}$/MoSi${}_{2}$ composites are fabricated by mechanical alloying in [8]. WSi${}_{2}$-reinforced MoSi${}_{2}$ composites are successfully prepared also by mechanical activation followed by in situ reactive spark plasma sintering of Mo, Si, and W elemental powders in [9]. The addition of W to the reactants leads to a finer microstructure than that obtained using pure MoSi${}_{2}$, resulting in a significant improvement of mechanical properties. The Vicker’s hardness of 20 vol % WSi${}_{2}$/MoSi${}_{2}$ is as high as 16.47 GPa. Nanocomposite of (Mo,W)Si${}_{2}$/WSi${}_{2}$ was synthesized via mechanical alloying (MA) and heat treatment in Ref. [10]. Increasing the milling time to 80 h followed by the post-annealing at 1000 ${}^{\circ }$C caused the complete formation of (Mo,W)Si${}_{2}$/WSi${}_{2}$ nanocomposite. MoSi${}_{2}$/WSi${}_{2}$ composites were successfully prepared by pressureless sintering from mechanically-assisted combustion synthesized powders in [11].

Motivated by the above-mentioned studies of MoSi${}_{2}$/WSi${}_{2}$ (nano)composites containing structurally and elastically very similar pair of materials crystallizing in the C11${}_{b}$ structure, we also assessed another pair of matching materials, TaSi${}_{2}$ and NbSi${}_{2}$, which are crystallizing in the C40 structure.

Finally, as a system with rather low interface energy, we studied nanocomposites formed by two phases from the Fe-Al binary system [12,13,14,15]. These materials are also considered as promising for elevated-temperature applications and as such they are intensively studied [16,17,18,19,20,21,22,23,24,25,26,27,28,29,30,31,32,33,34,35,36,37,38,39,40]. A sub-class of Fe-Al-based materials is represented by composites consisting of an ordered Fe${}_{3}$Al with the D0${}_{3}$ structure and a disordered Fe-Al solid solution with about 18-19 at.% Al. The existence of these composites can be experimentally proved using, for example, Mössbauer spectroscopy [41] or transmission electron microscopy (TEM) techniques. The latter are sensitive to anti-phase boundaries (APBs), which have a different character in Fe${}_{3}$Al and the Fe-Al phase [42,43,44,45].

## 2. Methods

Our quantum-mechanical calculations were performed within the framework of density functional theory [46,47] using the Vienna Ab initio Simulation Package (VASP) [48,49] and projector augmented wave (PAW) pseudopotentials [50,51]. When studying the transition-metal disilicides, the exchange and correlation energy was treated in the local density approximation (LDA) [52] but, in the case of phases containing the Fe and Al atoms, the generalized gradient approximation (GGA) parameterized by Perdew and Wang [53] (PW91) with the Vosko–Wilk–Nusair correction [54] was necessary to correctly reproduce the ground-state D0${}_{3}$ structure of Fe${}_{3}$Al. Regarding the MoSi${}_{2}$ and WSi${}_{2}$, we used a plane-wave energy cut-off of 400 eV and the k-point Monkhorst–Pack [55] meshes contained 20 × 20 × 2 (10 × 10 × 8) k-points in the case of 24-atom supercells modeling the superlattices with stacking along the [001] ([100] and [110]) directions. The calculations for TaSi${}_{2}$ and NbSi${}_{2}$ required the cut-off energy of 480 eV and the 12 × 12 × 4 k-point mesh in the case of 18-atom supercells. When computing Fe-Al-based nanocomposites, the cut-off energy was equal to 350 eV and the sampling of the Brillouin zone was done using Monkhorst–Pack grids with 10 × 10 × 6 k-points in the case of computational supercells containing 32 atoms. All calculations had an estimated error-bar of about 0.001 eV/atom. Finally, the second-order elastic constants were determined using the stress-strain method [56].

## 3. Results

**Figure 1:**
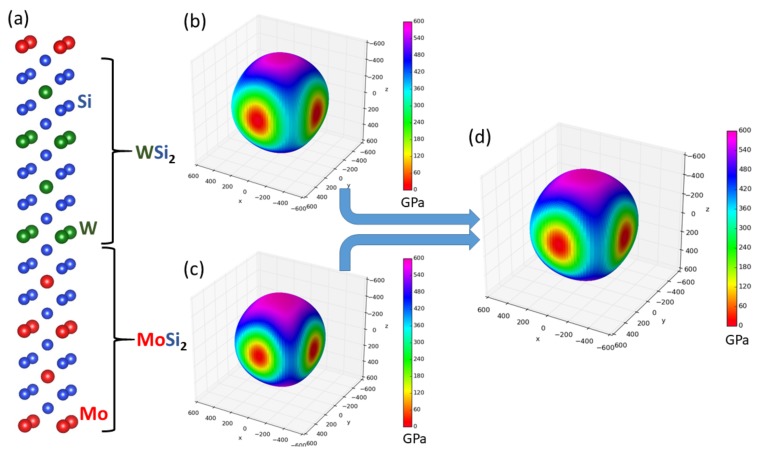
Visualization of a WSi${}_{2}$/MoSi${}_{2}$ nanocomposite (a superlattice) with the stacking along (and the interfaces perpendicular to) the [001] direction within the C11${}_{b}$ lattice (**a**) accompanied with directional dependences of the Young’s modulus of bulk WSi${}_{2}$ (**b**), bulk MoSi${}_{2}$ (**c**) and the composite WSi${}_{2}$/MoSi${}_{2}$ (**d**) formed out of them. The parts (**b**–**d**) were visualized by the SC-EMA [79,80,81] library (scema.mpie.de) based on ab initio computed elastic constants.

The first type of studied nanocomposite containing transition-metal disilicides is visualized in Figure 1a. WSi${}_{2}$ and MoSi${}_{2}$, which crystallize in the tetragonal C11${}_{b}$ structure, form a coherent nanocomposite where two conventional cells of each materials are stacked one on top of the other along the [001] direction (the interfaces are perpendicular to this direction) and alternate. It should be emphasized that, due to the periodic boundary conditions, which are applied to all nanocomposites in our calculations, the simulated nanocomposites form so-called superlattices [57,58,59,60,61,62,63,64,65,66,67,68,69,70,71,72,73,74,75,76,77,78] when the atomic planes continue from one phase into another.

As seen in Table 1, both disilicides have very similar lattice parameters *a* and *c* describing their tetragonal structure and also quite similar elastic properties. Our theoretical values are in excellent agreement with both experimental data and previous calculations. The elasticity of bulk phases is conveniently visualized in Figure 1b,c in the form of directional dependences of the Young’s modulus. The values for this composite with an equal amount of both phases are listed in Table 1 at the line for the composition (WSi${}_{2}$)${}_{4}$(MoSi${}_{2}$)${}_{4}$.

As a consequence of the similarity of the elastic constants of both constituents, the overall elastic properties of their nanocomposites are quite similar, too.

Next, we evaluated the interface energy of the nanocomposites according to the formula:

(1) $$\gamma \left({\left({\mathrm{WSi}}_{2}\right)}_{m}/{\left({\mathrm{MoSi}}_{2}\right)}_{n}\right)=\frac{E\left({\left({\mathrm{WSi}}_{2}\right)}_{m}/{\left({\mathrm{MoSi}}_{2}\right)}_{n}\right)-m\cdot E\left({\mathrm{WSi}}_{2}\right)-n\cdot E\left({\mathrm{MoSi}}_{2}\right)}{\left(2\cdot A\right)}$$

using the energy $E({\left({\mathrm{WSi}}_{2}\right)}_{m}/{\left({\mathrm{MoSi}}_{2}\right)}_{n})$ of the supercell modeling the nanocomposite ${\left({\mathrm{WSi}}_{2}\right)}_{m}/{\left({\mathrm{MoSi}}_{2}\right)}_{n}$, which contains *m* formula units of ${\mathrm{WSi}}_{2}$ and *n* formula units of ${\mathrm{MoSi}}_{2}$, the energies of bulk phases of each constituent $E({\mathrm{WSi}}_{2})$ and $E({\mathrm{MoSi}}_{2})$ and the area of the interface *A*. Importantly, very probably due to the similarity of both constituents, the interface energy was found to be ultra low, essentially zero within the error bar of our calculations, i.e., the energy differences in Equation (1) are smaller than 0.001 eV/atom and the interface energies are then smaller than about 0.005 J/m${}^{2}$.

**Figure 2:**
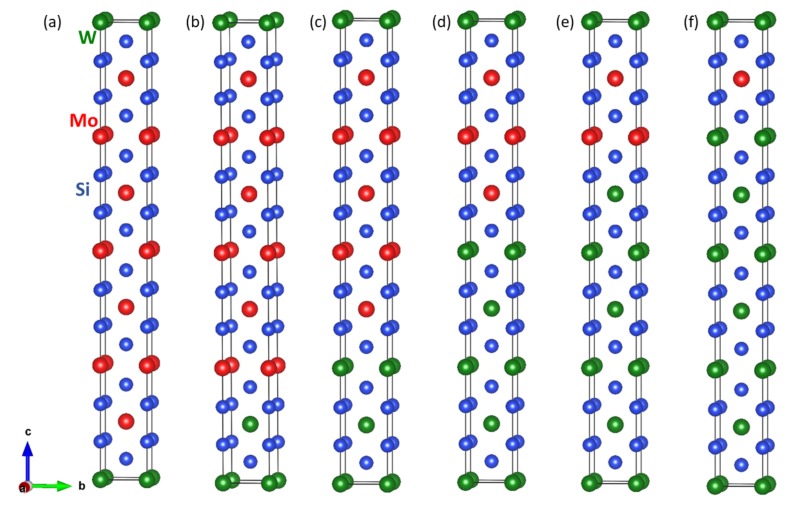
Schematic visualization of supercells modeling various nanocomposites with the stacking along (and the interfaces perpendicular to) the [001] direction. The mutual ratio of the constituents varies from 1:7, i.e., (WSi${}_{2}$)${}_{1}$(MoSi${}_{2}$)${}_{7}$, in the case of sub-figure (**a**), via 2:6 (**b**) and 3:5 (**c**) further for the opposite ratios 5:3 (**d**) and 6:2 (**e**) to 7:1, i.e., (WSi${}_{2}$)${}_{7}$(MoSi${}_{2}$)${}_{1}$, in the case of sub-figure (**f**).

To examine how the ultra-low interface energies depend on the width of the layers containing individual constituents within the nanocomposite as well as on the ratio of amount of both materials, we simulated a series of seven other superlattices with varying width of the constituents, as visualized in Figure 2. The calculated structural and elastic parameters are summarized in Table 1. The lattice parameter *a* increased quite monotonously from the value calculated for bulk MoSi${}_{2}$ to that obtained for bulk WSi${}_{2}$. The changes of the lattice parameter *c* are much smaller because the values found for bulk MoSi${}_{2}$ and WSi${}_{2}$ are only very slightly different. Rather monotonous changes appear also for the elastic constants with all of them increasing when increasing the amount of elastically stiffer WSi${}_{2}$. Importantly, all the studied nanocomposites have again ultra low interface energies, which are essentially zero within the error-bar of our calculations. Next, as seen in the Appendix, the ultra low interface energies were nearly independent of the crystallographic orientation of the interface.

**Figure 3:**
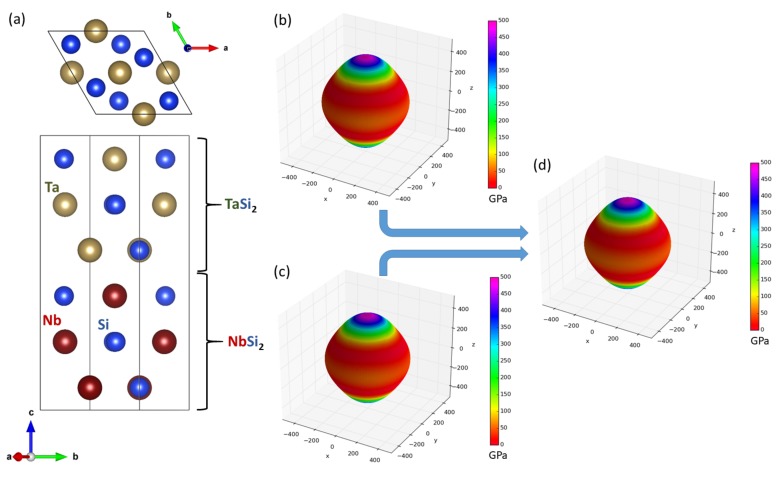
A top-view and a side-view of the computational supercell used in our calculations as a model of TaSi${}_{2}$/NbSi${}_{2}$ nanocomposite (a superlattice) with the stacking along (and the interfaces perpendicular to) the [0001] direction within the C40 lattice (**a**) accompanied with directional dependences of the Young’s modulus of bulk TaSi${}_{2}$ (**b**), bulk NbSi${}_{2}$ (**c**) and their composite TaSi${}_{2}$/NbSi${}_{2}$ (**d**).

After examining the WSi${}_{2}$/MoSi${}_{2}$ nanocomposites, which were experimentally reported to exist, we next drew our attention to another class of nanocomposites containing transition-metal disilicides, which were structurally and elastically very similar. The studied TaSi${}_{2}$ and NbSi${}_{2}$ crystallize in the hexagonal C40 structure and, therefore, we simulated a superlattice based on this structure (see Figure 3).

The nanocomposites have the interfaces perpendicular to the [0001] crystallographic direction. The lattice parameters *a* and *c* and elastic constants of both constituents in their bulk phases are summarized in Table 2. Our theoretical values are in an excellent agreement with both experimental data and previous calculations. Both disilicides have all the parameters very similar. The elasticity of bulk phases is conveniently visualized in Figure 3b,c in the form of directional dependences of the Young’s modulus. As the elastic properties are quite similar, the elasticity of the studied nanocomposite is not too different from that of the constituting phases (see Figure 3d). Importantly, the interface energy is ultra low for this superlattice, again zero within the error-bar of our calculations.

**Figure 4:**
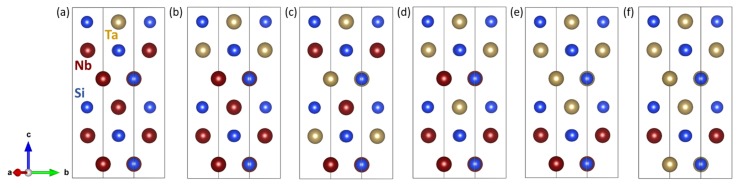
Schematic visualization of simulated NbSi${}_{2}$/TaSi${}_{2}$ nanocomposites with the stacking along (and the interfaces perpendicular to) the [0001] direction. The mutual ratio of the amount of constituents varies from 1:5, i.e., (NbSi${}_{2}$)${}_{1}$(TaSi${}_{2}$)${}_{5}$ in the case of (**a**) via 2:4 (**b**), 3:3 (**c**,**d**) to 4:2 (**e**) and 5:1, i.e., (NbSi${}_{2}$)${}_{5}$(TaSi${}_{2}$)${}_{1}$ in the case of (**f**). Variants shown in (**c**,**d**) have an equal amount of both constituting phases (similar to the case of Figure 3a) but a different arrangement of atomic layers. Consequently, there is a higher number of internal interfaces (6 and 4 in the case of (**c**,**d**), respectively) and the layers with different constituents have different widths.

Similar to the case of the WSi${}_{2}$/MoSi${}_{2}$ nanocomposites studied above, we examined how the ultra-low interface energies depend on the molar ratio of the constituting phases as well as on the width of the phases forming the superlattice (see Figure 4). We performed our calculations for a series of six other nanocomposites with different ratio of the TaSi${}_{2}$ and NbSi${}_{2}$ (see Figure 4a–f). Out of the six calculated superlattices shown in Figure 4, those shown in Figure 4c,d have the same ratio of the amount of both materials but a higher number of internal interfaces, six and four, respectively.

When evaluating the interface energies, they were again found to be ultra low, namely zero within the error-bar of our calculations. The lattice parameters *a* and *c* of the C40-based structure are concerned were rather monotonously increasing from their lower values in TaSi${}_{2}$ to higher values in NbSi${}_{2}$. In contrast to this trend, the elastic constants rather monotonously decreased from their higher values in TaSi${}_{2}$ to lower values in NbSi${}_{2}$.

**Figure 5:**
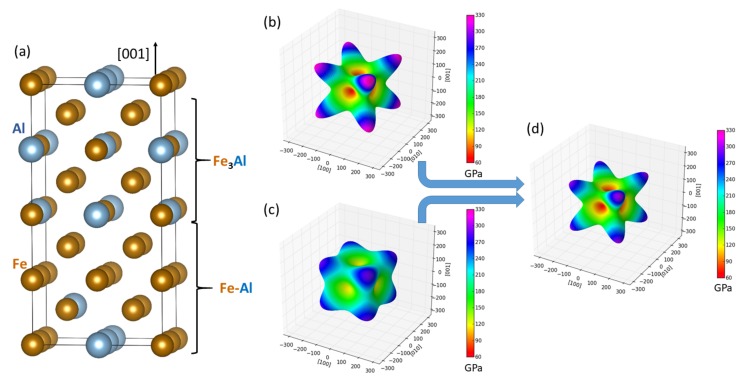
Visualization of a Fe${}_{3}$Al/Fe-Al nanocomposite (a superlattice) with the stacking along (and the interfaces perpendicular to) the [001] direction (**a**) accompanied with directional dependences of the Young’s modulus of bulk Fe${}_{3}$Al (**b**), bulk Fe-Al (**c**) and the nanocomposite Fe${}_{3}$Al/Fe-Al (**d**) formed out of them. Parts (**b**–**d**) were visualized by the SC-EMA [79,80,81] library (scema.mpie.de) based on ab initio computed elastic constants.

The last systems studied here is that containing two different phases from the binary iron-aluminium system, in particular, an ordered Fe${}_{3}$Al intermetallic compound crystallizing in the D0${}_{3}$ structure and a disordered solid solution of 18.75 at.% of Al with a body-centered cubic (bcc) ferromagnetic (FM) matrix, so-called α-or B2 phase. The structure of the former was is derived from the bcc lattice and, therefore, both materials structurally match each other. The studied nanocomposite is schematically visualized in Figure 5a. Fe${}_{3}$Al was modeled by a 16-atom conventional cell depicted in the upper part of in Figure 5a. The α-phase represents a challenge for quantum-mechanical calculations because it lacks any long-range periodicity. We used a 16-atom supercell with atoms distributed according to so-called special quasi-random structure (SQS) concept.

The nanocomposite was formed by stacking the Fe${}_{3}$Al on top of the α-phase along the [001] direction (the interfaces are perpendicular to this direction). In contrast to the above-discussed cases of superlattices formed by pairs of ordered transition-metal disilicides, when the both interfaces in the simulation supercell were identical, the supercells modeling the Fe-Al-based nanocomposites have different interfaces due to different distribution of atoms in the disordered α-phase. The interface energies were then averaged values related to the two interfaces. As another difference between the pairs of structurally and elastically nearly identical disilicides discussed above, the two constituting phases have clearly distinguishable elastic properties. Again, they are conveniently visualized in the form of directional dependences of the Young’s modulus in Figure 5b,c for the Fe${}_{3}$Al and the Fe-Al α-phase, respectively. The calculated values of elastic constants for Fe${}_{3}$Al compound are $C_{11}$ = 211 GPa, $C_{12}$ = 161 GPa and $C_{44}$ = 139 GPa. The elastic constants calculated for the disordered Fe-Al α-phase were projected onto a set of elastic constants possessing a cubic symmetry according to the rigorous mathematical theory by Moakher and Norris [87]. Similar concepts are often used in case of systems with any form of disorder (see, e.g., Refs. [59,88,89,90,91]). The resulting cubic-symmetry elastic constants are $C_{11}$ = 217 GPa, $C_{12}$ = 131 GPa and $C_{44}$ = 120 GPa. Both phases exhibit 〈001〉 directions as elastically soft and 〈111〉 directions as elastically hard (i.e., with the minimum and maximum values of the Young’s modulus, respectively). The Fe${}_{3}$Al is also apparently elastically more anisotropic. The overall elasticity of their composite is then shown in Figure 5d.

**Figure 6:**
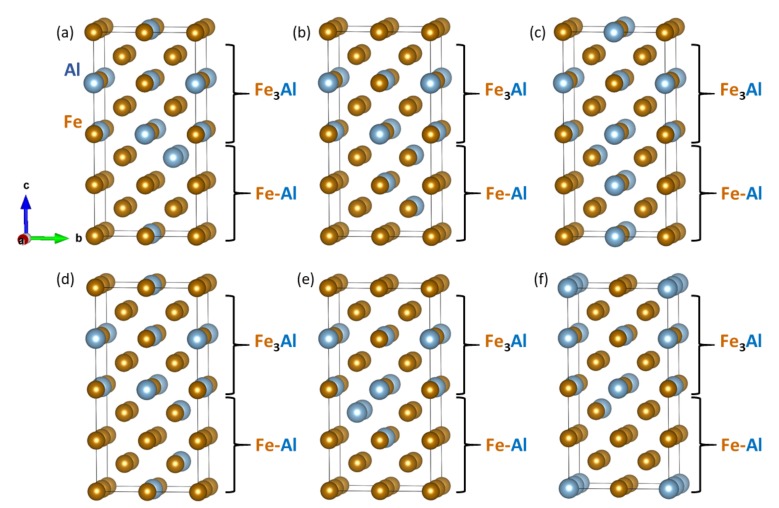
Schematic visualizations of different computed Fe${}_{3}$Al/Fe-Al nanocomposites. The computed variants shown in sub-figures (**a**–**f**) differ by the distribution of atoms in the disordered Fe-Al phase.

Importantly, the interface energy was found to be ultra low again, only 0.005 J/m${}^{2}$, which represents an energy difference appearing in Equation ((1)) smaller than 0.001 eV/atom, i.e., within the error-bar of our calculations. To determine an impact of distribution of atoms in the disordered Fe-Al α-phase on the interface energies, we performed a series of six additional calculations for supercells which have the same stoichiometry but differ in distributions of atoms in the Fe-Al α-phase (see Figure 6). In fact, the structure of coordination shells of atoms remain the same. For example, considering the Al atoms, their distribution in the part corresponding to the Fe-Al α-phase in the structural variants in Figure 6 are the same but the part of the supercell corresponding to the Fe-Al α-phase is either rotated and/or the atomic planes are permuted. As far as the latter process is concerned, if the atomic planes perpendicular to the [001] direction in the α-phase part of Figure 5a would be numbered 1, 2, 3, 4, then by a permutation is meant, e.g., an arrangement 2, 3, 4, 1. Importantly, if being in a single-phase bulk, such permutation of atomic planes within the periodically repeated cell or rotations of the whole cell would not change the energy because the position of the origin of coordinates (and the attached coordinate frame) can be arbitrarily shifted with respect to the crystal lattice.

The situation is, on the other hand, different in nanocomposites because the interface represents a reference point not existing in the single-phase bulk. The calculated energies are covering a broader range: 0.055 J/m${}^{2}$ (Figure 6a), 0.021 J/m${}^{2}$ (Figure 6b), 0.032 J/m${}^{2}$ (Figure 6c), 0.006 J/m${}^{2}$ (Figure 6d), 0.139 J/m${}^{2}$ (Figure 6e), and 0.014 J/m${}^{2}$ (Figure 6f). While this sensitivity on the atomic distribution is rather significant, the elastic properties of nanocomposites shown in Figure 6 are very similar. The computed values of elastic constants are summarized in Table 3.

**Figure 7:**
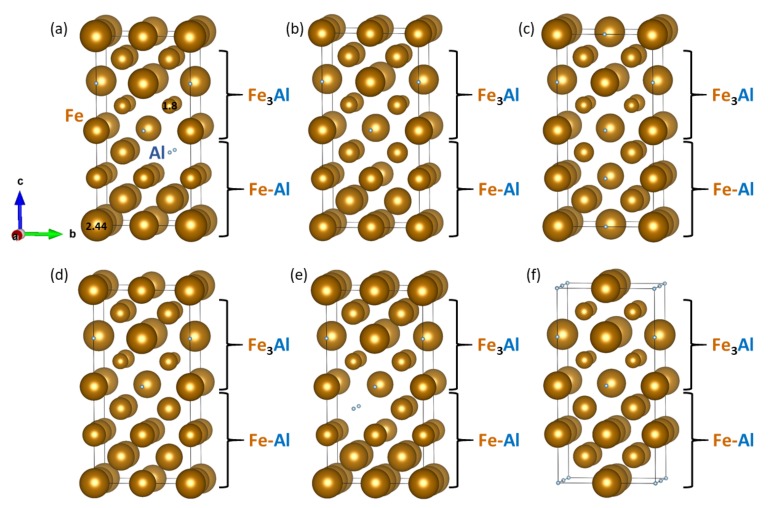
Calculated local magnetic moments of iron atoms (the magnitude is indicated by the diameter of the spheres representing the Fe atoms). The local magnetic moments shown in sub-figures (**a**–**f**) correspond to atomic distributions of nanocomposites visualized in sub-figures (**a**–**f**) of Figure 6, respectively.

As both phases appearing in the studied nanocomposites are magnetic, we further examined local magnetic moments of the iron atoms in configurations visualized in Figure 6a–f. The magnitude of local magnetic moments are shown in Figure 7 by the diameter of the spheres representing the Fe atoms. The lowest and the highest value (1.8 $\mu_{B}$ and 2.44 $\mu_{B}$) are explicitly mentioned in Figure 7a to demonstrate a scaling, by which the magnitude of the local magnetic moment is indicated by the diameter of the spheres. Importantly, the magnetic properties of the Fe atoms turned out to be very sensitive to the distribution of atoms (they are reduced when the Al atoms are nearby).

## 4. Discussion

The above-discussed ultra low interface energies in the MoSi${}_{2}$/WSi${}_{2}$ nanocomposites may also indicate that both constituents are prone to mixing even at the atomic level. Indeed, it seems that longer annealing times lead to formation of solid-solution phases rather than (nano)composites. For example, MoSi${}_{2}$/WSi${}_{2}$ powders are synthesized by means of self-propagating high temperature combustion in [92] but solid solutions of MoSi${}_{2}$/WSi${}_{2}$ and Mo${}_{5}$Si${}_{3}$/W${}_{5}$Si${}_{3}$ are found. In [93], it is also reported that it is hard to distinguish MoSi${}_{2}$ and WSi${}_{2}$ phases and (W,Mo)Si${}_{2}$ mainly in solid solution is found in Ref. [94]. The difficulties to distinguish MoSi${}_{2}$ and WSi${}_{2}$ Bragg peaks can be attributed to tetragonal MoSi${}_{2}$ and WSi${}_{2}$ phase having a long-range structure with very similar lattice constants (*a* is equal to 0.3202 nm and 0.3211, *c* amounts to 0.7855nm and 0.7835nm, respectively) [83]. It is also confirmed that MoSi${}_{2}$/WSi${}_{2}$ solid solution powder with nanometric (Mo,W)Si${}_{2}$ structure forms via combustion synthesis method from the mechanical activated powder mixture [95].

**Figure 8:**
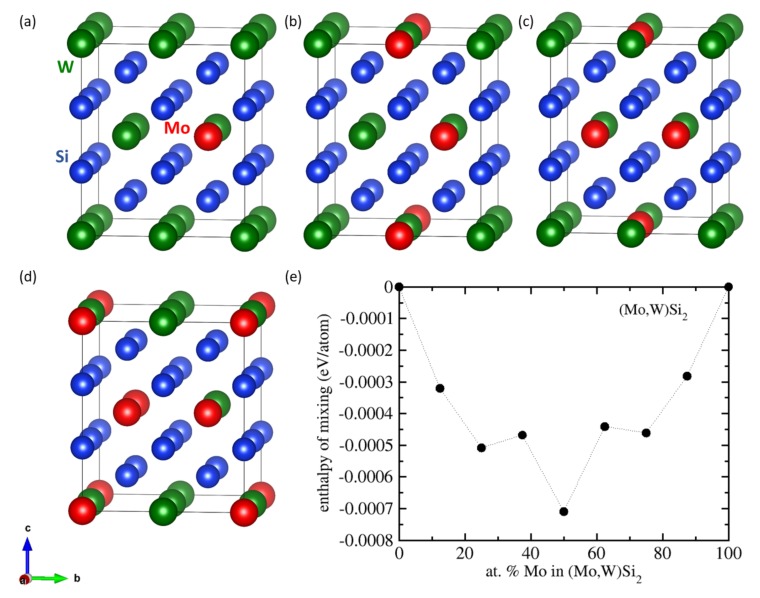
Schematic visualization of the supercells modeling random solid solutions of Mo and W within a C11${}_{b}$ lattice in the case of Mo:W ratio equal to 1:7 (**a**), 2:6 (**b**), 3:5 (**c**), and 4:4 (**d**) together with the correspondingenthalpies of mixing (**e**). The supercells for the Mo:W ratios equal to 5:3, 6:2 and 7:1 were obtained by swapping Mo and W atoms in the supercells shown in sub-figures (**a**–**c**). The atoms in the 32-atom supercells (2 × 2 × 1 multiple of 6-atom conventional cell of the C11${}_{b}$ structure) were distributed according to the special quasi-random structure (SQS) concept [96].

To test the scenario of a random solid solution of Mo and W within a C11${}_{b}$ lattice, we performed a series of calculations for supercells modeling these states (see Figure 8a–d). The corresponding enthalpies of mixing (evaluated with respect to the energy of MoSi${}_{2}$ and WSi${}_{2}$ as reference end-members) are shown in Figure 8e and all of them are between zero and −0.001 eV/atom, i.e., within the error-bar of our calculations and comparable to the energy differences obtained when simulating the MoSi${}_{2}$/WSi${}_{2}$ nanocomposites.

The above-discussed competition between formation of two-phase nanocomposites on the one hand and single-phase solid solutions on the other hand probably explains why a suitable preparation route is still being searched for in the case of TaSi${}_{2}$/NbSi${}_{2}$ nanocomposites when Nb solubility in TaSi${}_{2}$ extremely large [97]. Our results related to the TaSi${}_{2}$/NbSi${}_{2}$ nanocomposites are intended as a motivation for future studies of this interesting system.

## 5. Conclusions

We performed a first-principles study of structural, thermodynamic and elastic properties of nanocomposites exhibiting ultra low or low interface energies. As examples of systems with predominantly covalent interatomic bonds, we studied two combinations of transition-metal disilicides: (i) MoSi${}_{2}$/WSi${}_{2}$ nanocomposites with individual constituents crystallizing in the tetragonal C11${}_{b}$ structure; and (ii) TaSi${}_{2}$/NbSi${}_{2}$ with the two components crystallizing in the hexagonal C40 structure. The constituents within each pair of materials exhibit very similar structural and elastic properties and we obtained ultra low (nearly zero) interface energy for their nanocomposites (within the error bar of our calculations, i.e., about 0.005 J/m${}^{2}$). The interface energy was found to be nearly independent on the width of individual constituents within the nanocomposites and/or crystallographic orientation of the interfaces.

As an example of a magnetic system, a pair of metallic phases containing from the Fe-Al system with different atomic ordering was considered. In particular, we simulated coherent superlattices formed by an ordered Fe${}_{3}$Al intermetallic compound and a disordered Fe-Al phase with 18.75 at.% Al, the α-phase. Both constituents are structurally and elastically rather similar (but less than the two pairs of studied disilicides). To estimate the interface energy in the nanocomposite containing the disordered α-phase, which lacks a long-range periodicity, we simulated seven different distributions of atoms in the α-phase interfacing the Fe${}_{3}$Al intermetallic compound. The resulting interface energies were again either ultra low or low, from 0.005 to 0.139 J/m${}^{2}$. While the impact of atomic distribution on the elastic properties was found insignificant, the local magnetic moments of the iron atoms sensitively depended on the type and the distribution of surrounding atoms.

## Figures and Tables

**Table 1:** Calculated structural characteristics and elastic constants of bulk MoSi${}_{2}$ and WSi${}_{2}$ as well as of their composite MoSi${}_{2}$/WSi${}_{2}$ with the stacking along (and the interfaces perpendicular to) the [001] direction within the C11${}_{b}$ lattice. The computed values are complemented by experimental data from the literature. As far as the lattice parameters *a* and *c* of the C11${}_{b}$ structure are concerned, the values for the *c* lattice parameters of the nanocomposites are marked by the symbol of an asterisk * because they are divided by a factor of 4  to be compared with the values for the bulk unit cells of the individual constituents (bulk MoSi${}_{2}$ and WSi${}_{2}$). The simulated nanocomposites are shown in Figure 2 and, specifically for the equal amount of both phases, in Figure 1a. Experimental elastic constants of MoSi${}_{2}$ and WSi${}_{2}$ were taken from Refs. [82,83].

Composition	*a*	*c*	$C_{11}$	$C_{12}$	$C_{13}$	$C_{33}$	$C_{44}$	$C_{66}$
	(Å)	(Å)	(GPa)	(GPa)	(GPa)	(GPa)	(GPa)	(GPa)
MoSi${}_{2}$	3.176	7.781	428	125	101	537	208	207
	3.202 [83]	7.855 [83]	417.0 [82]	104.2 [82]	83.8 [82]	514.5 [82]	204.2 [82]	193.6 [82]
(WSi${}_{2}$)${}_{1}$/(MoSi${}_{2}$)${}_{7}$	3.178	7.782 *	433	126	101	542	210	209
(WSi${}_{2}$)${}_{2}$/(MoSi${}_{2}$)${}_{6}$	3.180	7.781 *	437	127	102	546	210	210
(WSi${}_{2}$)${}_{3}$/(MoSi${}_{2}$)${}_{5}$	3.181	7.781 *	440	127	102	551	211	212
(WSi${}_{2}$)${}_{4}$/(MoSi${}_{2}$)${}_{4}$	3.183	7.780 *	444	127	103	555	212	213
(WSi${}_{2}$)${}_{5}$/(MoSi${}_{2}$)${}_{3}$	3.185	7.780 *	447	127	103	560	212	214
(WSi${}_{2}$)${}_{6}$/(MoSi${}_{2}$)${}_{2}$	3.186	7.780 *	450	128	106	565	213	216
(WSi${}_{2}$)${}_{7}$/(MoSi${}_{2}$)${}_{1}$	3.188	7.780 *	453	128	106	570	213	217
WSi${}_{2}$	3.188	7.778	456	131	105	576	214	217
	3.211 [83]	7.835 [83]	442.8 [82]	121.7 [82]	81.0 [82]	552.3 [82]	211.6 [82]	217.5 [82]

**Table 2:** Calculated structural characteristics and elastic constants of bulk TaSi${}_{2}$ and NbSi${}_{2}$ as well as of their nanocomposites TaSi${}_{2}$/NbSi${}_{2}$ with the stacking along (and the interfaces perpendicular to) the [0001] direction within the C40 lattice. The computed values are complemented by both experimental data as well as by other theoretical results from literature. As far as the lattice parameters *a* and *c* of the C40 structure are concerned, the values of the lattice parameter *c* for the nanocomposites are marked by an asterisk * because they are divided by factor of 2  to be compared with the values for the bulk unit cells of the individual constituents (bulk TaSi${}_{2}$ and NbSi${}_{2}$). Experimental elastic constants of TaSi${}_{2}$ and NbSi${}_{2}$ were taken from Ref. [84] and theoretical ones from Refs. [85,86].

Composition	*a*	*c*	$C_{11}$	$C_{12}$	$C_{13}$	$C_{33}$	$C_{44}$
	(Å)	(Å)	(GPa)	(GPa)	(GPa)	(GPa)	(GPa)
TaSi${}_{2}$	4.736	6.530	394	85	101	487	143
	4.77 [84]	6.55 [84]	375.3 [84]	78.4 [84]	90.1 [84]	467.7 [84]	143.7 [84]
	4.731 [85]	6.501 [85]	392.2 [85]	78.3 [85]	98.2 [85]	484.6 [85]	148.8 [85]
	–	–	351.0 [86]	84.0 [86]	73.0 [86]	461.0 [86]	123.0 [86]
(NbSi${}_{2}$)${}_{1}$/(TaSi${}_{2}$)${}_{5}$—Figure 4a	4.739	6.534 *	392	84	99	483	143
(NbSi${}_{2}$)${}_{2}$/(TaSi${}_{2}$)${}_{4}$—Figure 4b	4.742	6.539 *	390	83	98	479	142
(NbSi${}_{2}$)${}_{3}$/(TaSi${}_{2}$)${}_{3}$—Figure 3a	4.745	6.543 *	387	82	96	475	142
(NbSi${}_{2}$)${}_{3}$/(TaSi${}_{2}$)${}_{3}$—Figure 4c	4.745	6.543 *	387	82	96	476	142
(NbSi${}_{2}$)${}_{3}$/(TaSi${}_{2}$)${}_{3}$—Figure 4d	4.745	6.543 *	388	82	96	476	142
(NbSi${}_{2}$)${}_{4}$/(TaSi${}_{2}$)${}_{2}$—Figure 4e	4.748	6.547 *	385	81	95	472	142
(NbSi${}_{2}$)${}_{5}$/(TaSi${}_{2}$)${}_{1}$—Figure 4f	4.751	6.551 *	383	80	94	469	141
NbSi${}_{2}$	4.754	6.555	380	79	92	465	141
	4.79 [84]	6.59 [84]	380.2 [84]	75.9 [84]	88.3 [84]	468.0 [84]	145.3 [84]
	4.747 [85]	6.529 [85]	378.9 [85]	73.0 [85]	90.2 [85]	462.5 [85]	144.6 [85]
	–	–	344.0 [86]	85.0 [86]	69.0 [86]	456.0 [86]	115.0 [86]

**Table 3:** Calculated elastic constants of Fe${}_{3}$Al/Fe-Al nanocomposites with the stacking along (and the interfaces perpendicular to) the [001] direction. The nanocomposites are visualized in Figure 6.

Variant	$C_{11}$	$C_{12}$	$C_{13}$	$C_{22}$	$C_{23}$	$C_{33}$	$C_{44}$	$C_{55}$	$C_{66}$
	(GPa)	(GPa)	(GPa)	(GPa)	(GPa)	(GPa)	(GPa)	(GPa)	(GPa)
Fe${}_{3}$Al/Fe-Al Figure 6a	188	143	134	201	139	199	120	126	124
Fe${}_{3}$Al/Fe-Al Figure 6b	186	134	142	185	141	199	123	123	123
Fe${}_{3}$Al/Fe-Al Figure 6c	197	146	145	196	145	184	129	129	124
Fe${}_{3}$Al/Fe-Al Figure 6d	200	151	143	199	143	200	125	125	122
Fe${}_{3}$Al/Fe-Al Figure 6e	183	135	138	189	136	202	117	124	123
Fe${}_{3}$Al/Fe-Al Figure 6f	175	141	137	189	145	200	121	126	127

**Figure A1:**
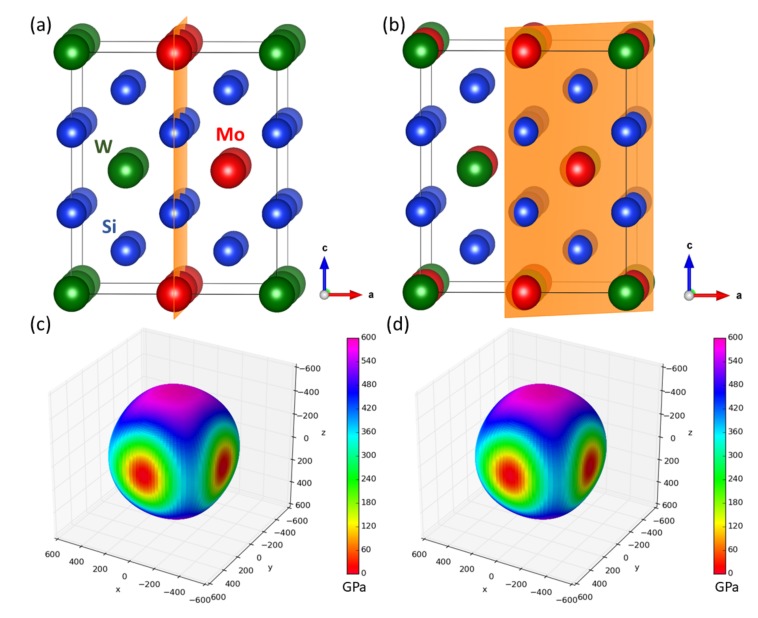
Schematics of computational supercells of MoSi${}_{2}$/WSi${}_{2}$ nanocomposites (superlattices) with the stacking along (and the interfaces perpendicular to) the [010] (**a**) and [110] (**b**) directions within the C11${}_{b}$ lattice, respectively. The interface planes are marked by orange color. The calculated elastic constants for these superlattices are shown as directional dependences of the Young’s modulus for the composite with the [010] stacking direction (**c**) as well as that with the [110] stacking direction (**d**). Parts (**c**,**d**) were visualized by the SC-EMA [79,80,81] library (scema.mpie.de) based on ab initio computed elastic constants.

## Appendix A

To analyze the dependence of the ultra-low interface energies in the MoSi${}_{2}$/WSi${}_{2}$ nanocomposites on the crystallographic orientation of the interface, we computed properties of two other superlattices with the interfaces perpendicular to the [010] and [110] directions, respectively. The corresponding supercells modeling these nanocomposites are shown in Figure A1a,b, respectively. The elastic properties of these superlattices are visualized in the form of directional dependences of the Young’s modulus in Figure A1c,d. It is obvious that they are again very similar to each other and also very similar to that of the superlattice with the interfaces perpendicular to the [001] direction.
